# Treatment of superior vena cava syndrome using AngioJet™ thrombectomy system

**DOI:** 10.1186/s42155-019-0071-3

**Published:** 2019-08-14

**Authors:** Amit Ramjit, Jesse Chen, Marcus Konner, Elliot Landau, Noor Ahmad

**Affiliations:** 1 Zucker School of Medicine at Hofstra, Northwell at Staten Island University Hospital, Staten Island, NY USA; 2 Touro College, College of Osteopathic Medicine, New York, NY USA

**Keywords:** SVC syndrome, AngioJet, Rheolytic Thrombectomy

## Abstract

**Background:**

Superior vena cava syndrome is a relatively rare presentation in which diminished venous return to the heart produces congestion of the neck, face and upper extremities. Typically, a mediastinal mass produces external compression on the superior vena cava and reduces venous return. However, superior vena cava syndrome can present acutely in the setting of vena cava thrombosis. Multiple scoring systems are available to assist clinicians with appropriate timing of interventions for SVC syndrome. When specific criteria are met, endovascular intervention can be beneficial to patients to prevent rapid deterioration.

**Case presentation:**

A 75-year-old female with no significant past medical history presented to the emergency department with increased facial swelling, nausea and vomiting which began the night prior to presentation. The patient underwent a CT chest which revealed a 3.2 × 3.0 × 3.8 cm spiculated mass compressing the right main bronchus and right pulmonary artery. The patient was intubated and interventional radiology was consulted. The patient underwent venography which showed extensive thrombosis of the innominate veins. Rheolytic thrombectomy with AngioJet™ was performed to alleviate clot burden and minimize risk of secondary pulmonary embolism. Kissing stents were placed in the bilateral innominate veins to maintain patency after thrombectomy. After the procedure, the patient was successfully extubated and had near complete resolution of facial swelling approximately 12 h post procedure. A follow up venogram performed on post procedure day 4 showed patent bilateral subclavian, innominate, and internal jugular veins as well as a patent superior vena cava.

**Conclusions:**

Acute occlusion of superior vena cava can present with life threatening symptoms such as loss of airway. AngioJet™ thrombectomy is another tool available to interventional radiologists when a patient’s clinical condition necessitates treatment.

## Background

Superior vena cava (SVC) syndrome occurs when drainage from the head and upper extremities becomes significantly reduced, leading to venous congestion. Symptoms include facial plethora, swelling of the neck and extremities, dyspnea and visual disturbances. When symptoms are rapidly progressive, the etiology is most commonly an underlying malignancy, such as a primary pulmonary tumor or metastatic disease (Lauten et al. 2010). When a patient presents with acute SVC syndrome and has critical symptoms such as airway compromise, interventional radiologists are frequently consulted for management. To manage patients successfully in short term, options include angioplasty, mechanical thrombectomy, thrombolysis and venous stenting (Dib and Hennebry 2012). With our patient, we chose to perform AngioJet™ (Boston Scientific, Marlborough, MA) rheolytic thrombectomy in conjunction with kissing stents to alleviate acute venous congestion and decrease thrombotic burden that could become pulmonary emboli (Ptohis et al. 2015). This method provides immediate decompression of the obstruction and maintains patency of the superior vena cava when external compression from a tumor was likely the cause of thrombosis.

## Case presentation

**Fig. 1:**
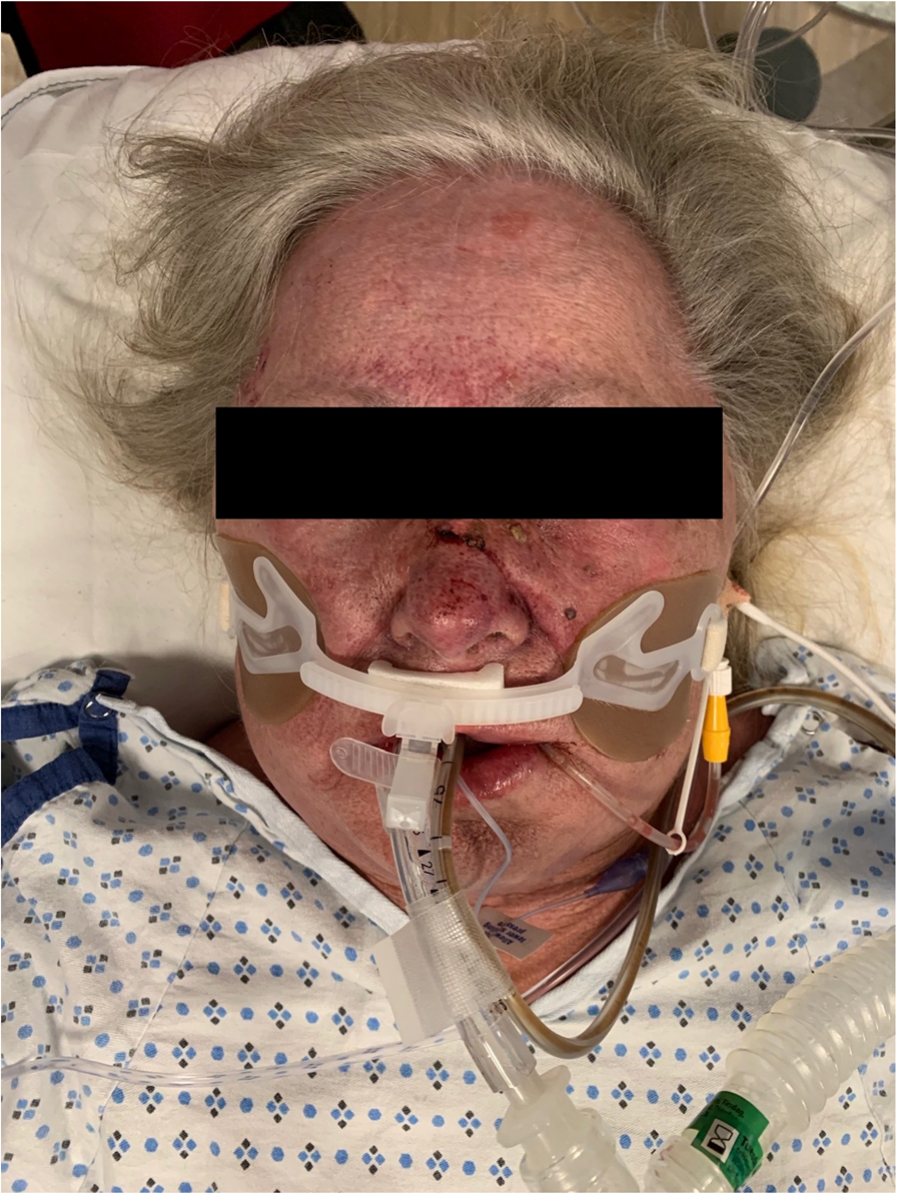
Patient’s clinical appearance after being intubated. Her face is engorged from venous obstruction

Our patient presented to the emergency department with the chief complaint of nausea and vomiting. The patient started to develop significant facial swelling on the evening prior to presentation. The patient began vomiting multiple times and had a possible brief syncopal episode while vomiting and lacerated the bridge of her nose on the commode. On the subsequent morning, the patient’s son noticed the skin changes and facial swelling. The patient was brought to the emergency department and underwent a CT of the chest, abdomen and pelvis. This study showed a right upper lobe spiculated mass measuring up to 3.8 cm with compression on the right mainstem bronchus and right pulmonary artery. Filling defects were seen in the innominate veins with multiple venous collateral vessels, suggestive of SVC syndrome. In addition, subsegmental pulmonary emboli and invasion of the SVC by the mass were visualized. On hospital day 1, the patient was admitted to the ICU, started on a heparin infusion and intubated given concerns of airway compromise (Fig. 1). Based on the combination of changes in mentation, facial swelling and lip edema, the patient was given a score of 4 on the Kishi scoring system for superior vena cava syndrome. Interventional radiology was consulted for endovascular management of SVC syndrome.

**Fig. 2:**
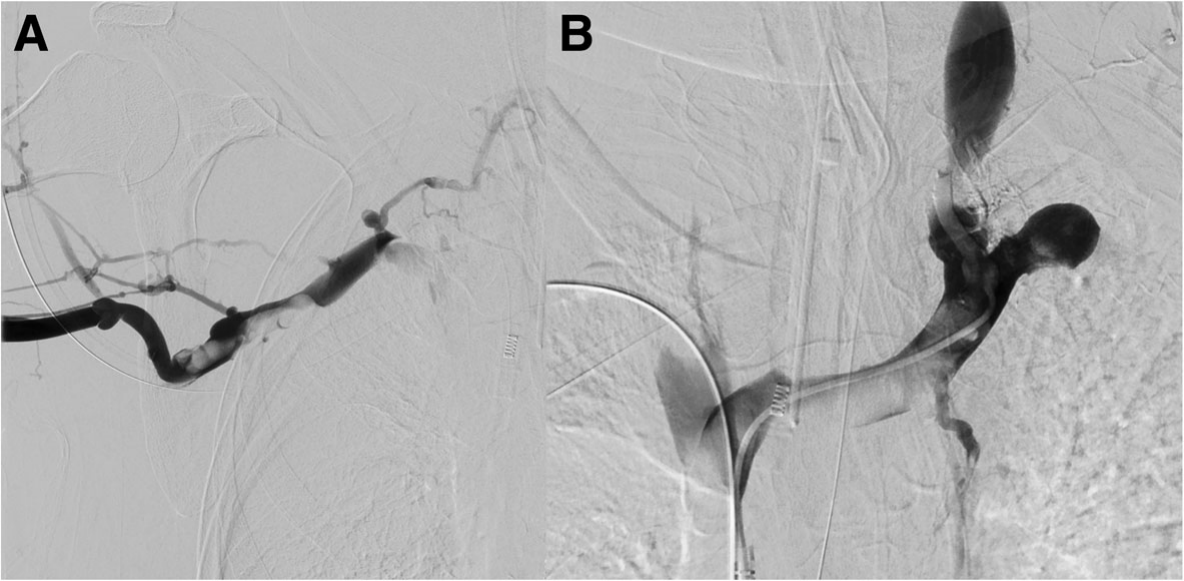
Initial venography shows extensive thrombus within the right subclavian (**a**) and superior vena cava(**b**)

Family members provided consent for image–guided thrombectomy and stent placement. The patient was brought to the angiography suite intubated on hospital day 2. Preliminary ultrasound demonstrated a completely thrombosed right internal jugular and partially thrombosed left internal jugular veins. Due to the completely thrombosed right internal jugular vein, ultrasound guided access of the right basilic vein using Seldinger technique was obtained and an 8 French vascular sheath was placed. Over a 0.035 Glidewire, a 5 French Berenstein catheter was advanced into the innominate veins. Venography demonstrated extensive thrombus within the right subclavian vein and superior vena cava (Fig. 2). In addition, there was high-grade narrowing at the cavoatrial junction secondary to extrinsic compression from the known right upper lobe pulmonary mass. The Berenstein catheter was negotiated beyond the cavoatrial junction narrowing into the infrarenal IVC over the Glidewire. The Glidewire was then exchanged for a super stiff exchange length Amplatz.

Due to the presence of thrombus within the bilateral internal jugular veins, a decision was made to access the right common femoral vein to allow for bilateral internal jugular vein intervention. Under ultrasound guidance, the right common femoral vein was punctured and an 8 French, 70 cm vascular sheath was placed using standard Seldinger technique with distal tip at the SVC-atrial junction. A 5 French Berenstein catheter was advanced beyond the narrowing of the cavoatrial junction and the bilateral internal jugular veins were successfully selected. Venography was performed which demonstrated the narrowing at the cavoatrial junction as well as extensive thrombus within the SVC, right internal jugular, left subclavian, left innominate, and left internal jugular veins with multiple collateral vessels draining centrally. The compression extended to the origin of the innominate vein. The Glidewire was exchanged for an Amplatz wire, with distal tip positioned within the left internal jugular vein. Due to the extensive thrombus burden and pre-existing pulmonary compromise, the decision was made to perform pharmacothrombolysis prior to establishing outflow.

At our institution, the EKOS infusion catheter (EKOS Corporation, Bothell, WA) and AngioJet™ ZelanteDVT thrombectomy system (Boston Scientific, Marlborough, MA) are available for clinical use. Catheter directed thrombolysis has high clinical success and long-term patency. The AngioJet™ thrombectomy catheter system was chosen because it facilities high dose thrombolytic infusion in a short period of time in the power pulse mode. This allows rapid dubulking of thrombus to prevent iatrogenic pulmonary emboli.

The existing Berenstein catheter in the right internal jugular vein from right common femoral vein access was exchanged for the 8 French AngioJet™ ZelanteDVT catheter. The 8 French AngioJet™ ZelanteDVT infusion catheter was advanced into the bilateral internal jugular veins and thrombolysis was performed using 10 mg tPA diluted in 100 mL of normal saline using the power pulse mode. During thrombolysis, the patient developed a brief episode of brachycardia which resolved upon pausing the AngioJet™ system. When the patient returned to a sinus rhythm, thrombolysis was continued for a total of approximately 3 min. Repeat angiography demonstrated decrease in thrombus burden and the AngioJet™ catheter was removed.

To establish outflow and overcome the extrinsic compression from the pulmonary mass, the decision was made to place kissing stents from the SVC into the bilateral brachiocephalic veins. Over the right basilic vein Amplatz wire, a 12 mm × 4 cm uncovered stent (Bard, New Providence, NJ) was placed across the cavoatrial narrowing along the length of the right innominate vein. Via the right common femoral vein, a 12 mm × 6 cm stent was placed across the cavoatrial narrowing and extending into the left subclavian vein. The stents were subsequently dilated using a 14 mm × 6 cm angioplasty balloon. Post stenting venography demonstrated persistent narrowing above the level of the right innominate vein. A second, 12 mm × 4 cm uncovered stent was advanced over the right basilic vein Amplatz wire. Final venography demonstrated improved flow through the bilateral jugular veins. The procedure was completed successfully and the patient returned to the ICU. The heparin infusion was continued post procedurally.

**Fig. 3:**
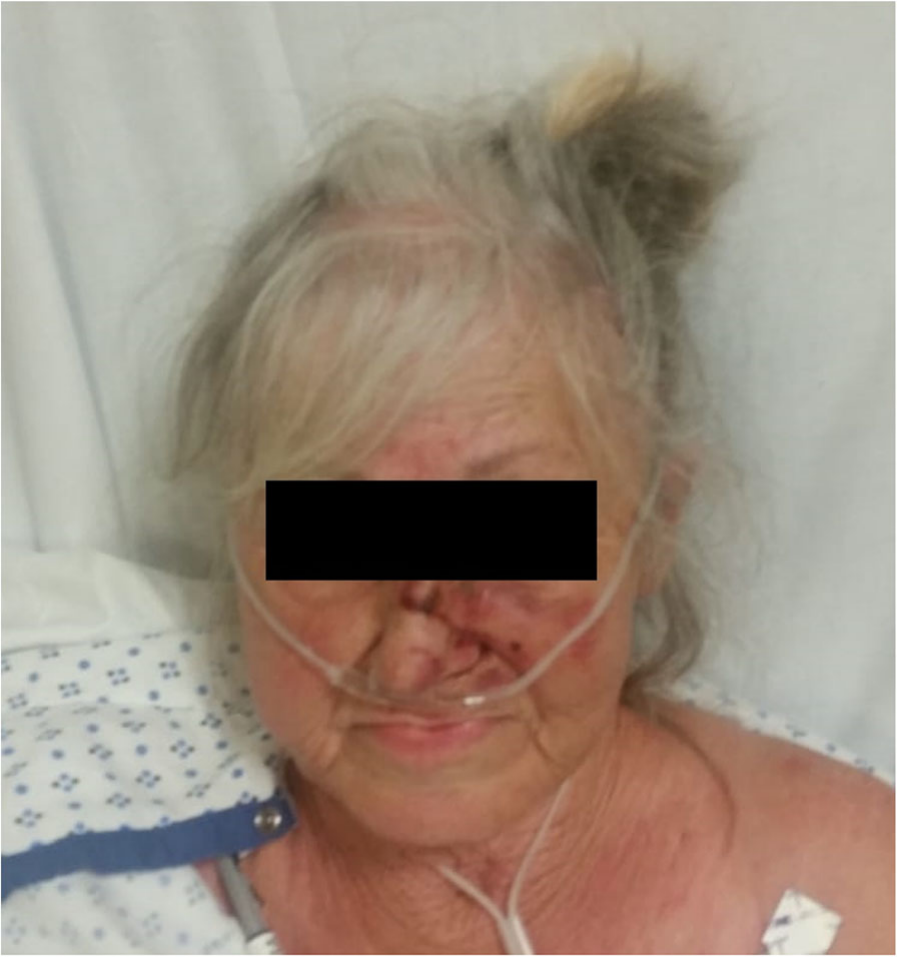
The patient’s appearance after extubation on post procedure day 1

**Fig. 4:**
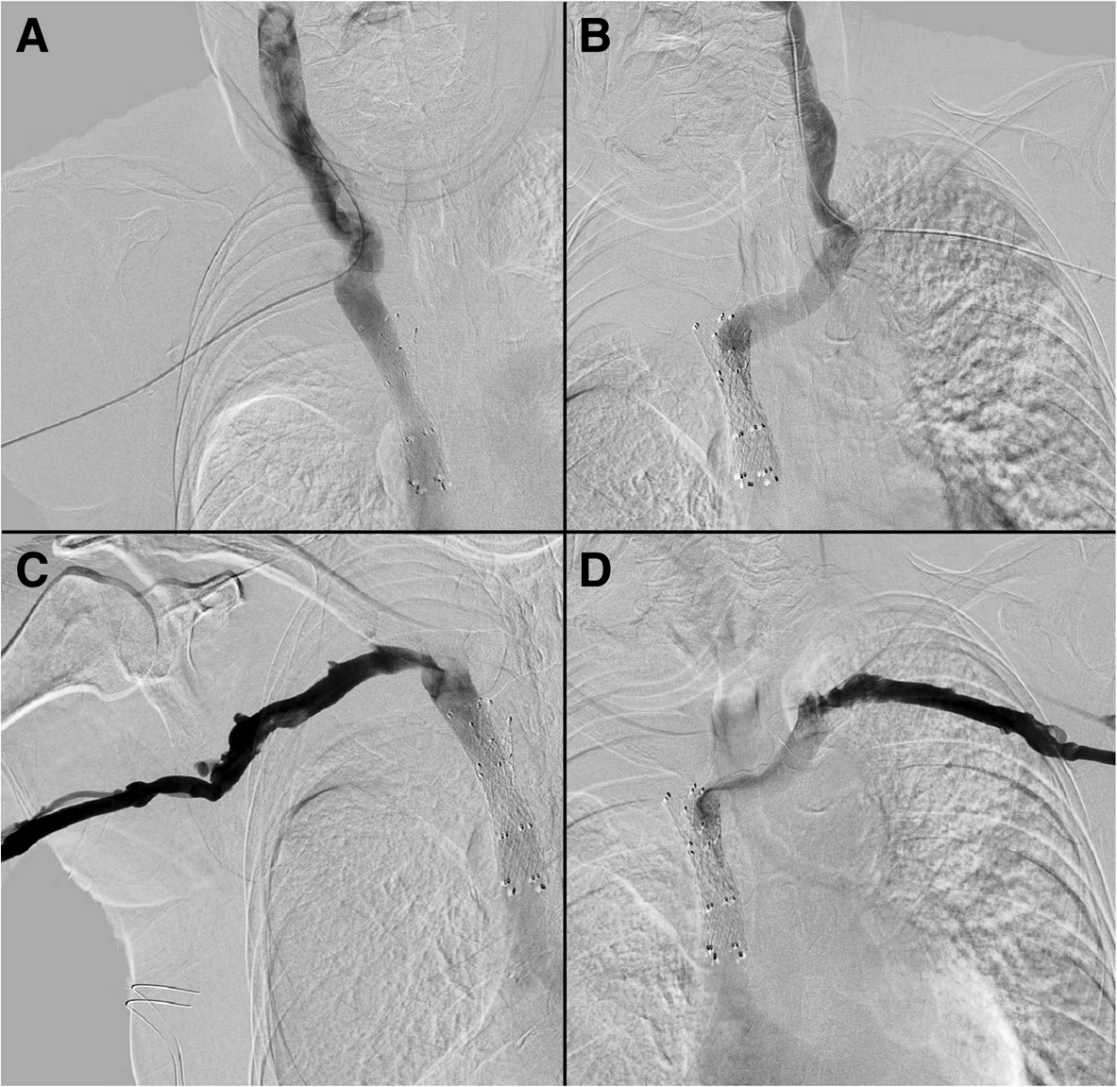
Venography shows patient right internal jugular (**a**), left internal jugular (**b**), right subclavian (**c**) and left subclavian (**d**) veins

On hospital day 3, the patient had a significant reduction of facial edema and was extubated (Fig. 3). The patient continued to slowly improve clinically. The patient returned to interventional radiology for venography on post procedure day 4. Venography from the left basilic and internal jugular veins demonstrated stent patency without residual thrombus. Venography from the right basilic and internal jugular veins demonstrated stent patency with minimal, non-occlusive thrombus within the right internal jugular vein (Fig. 4). Outpatient endobronchial ultrasound biopsy records became available which demonstrated squamous cell carcinoma for the tumor adjacent to the right main bronchus. The patient was transitioned to apixaban and discharged on hospital day 10.

## Discussion

Superior vena cava syndrome affects approximately 15,000 patients per year (Seligson and Surowiec 2019). The majority of cases are secondary to metastatic lung cancer with line associated thrombosis from central venous catheters and cardiac pacemakers as iatrogenic sources of SVC syndrome (Straka et al. 2016). The endovascular approach can achieve rapid symptomatic relief in benign and malignant causes of SVC syndrome. However, the long term results following stent placement are unknown and rethrombosis rates can be significant. Despite these potential shortcomings, endovascular techniques are the accepted first-line therapy for SVC syndrome (Kalra et al. 2018).

Several treatment options exist in the literature. Catheter directed thrombolysis (CDT) was first introduced as a modality to treat peripheral thrombi and has since been used to treat extensive thrombus within the superior vena cava (Ghanavati et al. 2017; Dotter et al. 1974). However, most examples in the literature combine percutaneous transarterial (PTA) techniques using 10 to 16 mm angioplasty balloons with stents (Kalra et al. 2018). The largest case series available in the literature for malignant SVC syndrome showed approximately 79% clinical patency with CDT and stent placement over an average 7 month follow up (Kee et al. 1998). Small perforations can occur which can be managed with prolonged balloon inflation. If SVC rupture occurs, pericardial tamponade must be rapidly diagnosed and treated with ultrasound guided pericardial drainage as necessary.

The first iteration of AngioJet™ was approved in 1996 for arteriovenous access graft thrombectomy as an alternative to traditional Fogarty-type catheters (US Food and Drug Administration 1996). Rheolytic thrombolysis uses a high-pressure infusion pump forcing saline through a 5 French rapid exchange catheter. The system’s catheter has a double lumen; a smaller one to run saline distally and a larger one to collect aspirated material. The saline solution exits the tip at approximately 500 km/h to create a circumferential zone of depression by the Bernoulli effect which crumples and aspirates thrombus (Petronio et al. 2010). Rheolytic thrombectomy has since been widely adapted for percutaneous coronary intervention (PCI) and treatment for lower extremity deep vein thrombosis (Antoniucci et al. 2004; Garcia et al. 2015; Leung et al. 2015; Elgendy et al. 2017).

A limited number of cases of rheolytic thrombectomy for SVC syndrome are available in the literature. AngioJet™ was used to treat SVC syndrome following orthotopic heart transplant for hypoplastic left heart syndrome. This pediatric patient underwent primary end-to-end anastomosis at the vena cava because of a previously performed Fontan procedure. Anastomosis at the vena cava predisposed the patient to acute stenosis at the vena cava. The patient was treated with rheolytic thrombectomy for approximately 47 s and 10 mm balloon angioplasty. The patient was started on warfarin therapy for 2 weeks and did not shows signs of recurrent obstruction or other thrombotic events at 6 months (Sessions et al. 2016).

After an extensive literature search, our case represents the second published use of AngioJet™ thrombectomy to treat SVC syndrome. Similar to the previously published case, our patient had no residual symptoms of SVC syndrome following the procedure. The patient established follow up at an outside institution for continued oncologic care.

## Conclusion

Rheolytic thrombectomy for SVC syndrome is currently an off-label indication for AngioJet™. When faced with potentially life-threatening conditions, the interventional radiologist should consider all options available for treatment. Investigational data, reports within the literature and clinical experience should guide patient specific treatment. Our experience with AngioJet™ demonstrates its applicability for SVC syndrome with short term technical and clinical success.

## Data Availability

Not applicable.

## Abbreviations

CDT: catheter directed thrombolysis
PCI: percutaneous coronary intervention
PTA: percutaneous transarterial
SVC: superior vena cava
